# Simultaneous Detection of Ebola Virus and Pathogens Associated With Hemorrhagic Fever by an Oligonucleotide Microarray

**DOI:** 10.3389/fmicb.2021.713372

**Published:** 2021-07-30

**Authors:** Wenwu Yao, Zhangnv Yang, Xiuyu Lou, Haiyan Mao, Hao Yan, Yanjun Zhang

**Affiliations:** Zhejiang Provincial Center for Disease Control and Prevention, Hangzhou, China

**Keywords:** Ebola virus, hemorrhagic fever, microarray, detection, pathogen

## Abstract

Ebola virus infection causes severe hemorrhagic fever, and its mortality rates varied from 25 to 90% in the previous outbreaks. The highly infectious and lethal nature of this virus highlights the need for reliable and sensitive diagnostic methods to distinguish it from other diseases present with similar clinical symptoms. Based on multiplex polymerase chain reaction (PCR) and oligonucleotide microarray technology, a cost-effective, multipathogen and high-throughput method was developed for simultaneous detection of Ebola virus and other pathogens associated with hemorrhagic fever, including Marburg virus, Lassa fever virus, Junin virus, Machupo virus, Rift Valley fever virus, Crimean-Congo hemorrhagic fever virus, malaria parasite, hantavirus, severe fever with thrombocytopenia syndrome virus, dengue virus, yellow fever virus, Chikungunya virus, influenza A virus, and influenza B virus. This assay had an excellent specificity for target pathogens, without overlap signal between the probes. The limit of detection was approximately 10^3^ pathogen copies/μl. A total of 60 positive nucleic acid samples for different pathogens were detected, a concordance of 100% was observed between microarray assay and real-time PCR analysis. Consequently, the described oligonucleotide microarray may be specific and sensitive assay for diagnosis and surveillance of infections caused by Ebola virus and other species of hemorrhagic fever pathogens.

## Introduction

Ebola virus disease (EVD), formerly known as Ebola hemorrhagic fever, is a severe, often fatal illness in humans. The first outbreaks of Ebola virus were documented in Sudan and the Democratic Republic of Congo in 1976 (Gire et al., 2014). Since its reemergence in 2013, Ebola virus has infected more than 32,000 people in Africa, resulting in more than 13,000 deaths (Tong et al., 2015; Maxmen, 2020). Similar to other viral hemorrhagic fever (VHF), following an incubation period of EVD, infected patients commonly develop non-specific flulike symptoms of fever, chills, malaise, and myalgia. The subsequent signs and symptoms indicate multisystem involvement and include systemic, gastrointestinal, respiratory, vascular, and neurological manifestations. Haemorrhagic manifestations occur during the peak of the illness and include petechiae, ecchymoses, uncontrolled oozing from venepuncture sites, mucosal hemorrhages, and postmortem evidence of visceral haemorrhagic effusions (Feldmann and Geisbert, 2011). The typical clinical symptoms of VHF include fever and bleeding disorders which are often accompanied with headache, muscle joint pain and convulsions which can progress to severe disseminated intravascular coagulation (DIC), central nervous system damage, shock, and even death (Marty et al., 2006). Many people throughout the world are exposed to the threat of hemorrhagic fever viruses, including Ebola virus, Marburg virus, Lassa fever virus, Junin virus, Machupo virus, Rift Valley fever virus, Crimean-Congo hemorrhagic fever virus, hantavirus, severe fever with thrombocytopenia syndrome virus, dengue virus, yellow fever virus, Chikungunya virus, etc. Most of the infections lacking specific clinical features at early stage of the disease must rely on laboratory diagnostic methods for identification, as the symptoms are similar to influenza or malaria.

There is a persistent need for cost-effective and reliable approaches for distinguishing Ebola virus from other pathogens associated with hemorrhagic fever. Currently, the laboratory methods for detecting Ebola virus include polymerase chain reaction (PCR), enzyme-linked immunosorbent assay (ELISA), indirect immunofluorescence assay (IFA), immunohistochemistry, virus isolation, etc. (Huang et al., 2014; Liu et al., 2015; Martin et al., 2015). However, the major disadvantage of each method mentioned above is that each assay can only test one infectious agent of the specimens. Although the multiple PCR can simultaneously detect more than one pathogen in one reaction, the quantity of pathogen is limited and this method cannot meet the requirement of high throughput, further it is also time consuming, costly, and requires large amount of samples.

Oligonucleotide microarray, a highthroughput technology that is accurate, speedy, and low-cost has been used for disease diagnosis, pathogenic microorganism detection, gene expression analysis, single-nucleotide polymorphism (SNP) detection, and genome sequencing (Leski et al., 2009; Chen et al., 2011; Kolquist et al., 2011; Mazzatti et al., 2012; Zhang et al., 2013; Katoski et al., 2015; Hardick et al., 2016). In this study, we report the establishment of an oligonucleotide microarray method for simultaneous identification of 16 pathogens associated with hemorrhagic fever, including Zaire ebolavirus (ZEBOV), Sudan ebolavirus (SEBOV), Marburg virus (MARV), Lassa fever virus (LFV), Junin virus (JUNV), Machupo virus (MACV), Rift Valley fever virus (RVFV), Crimean-Congo hemorrhagic fever virus (CCHFV), malaria parasite (MP), hantavirus (HV), severe fever with thrombocytopenia syndrome virus (SFTSV), dengue virus (DENV), yellow fever virus (YFV), Chikungunya virus (CHIKV), influenza A virus (FluA), and influenza B virus (FluB).

## Materials and Methods

### Specimen Collection and Processing

Serum or plasma specimens, blood samples, and clinical throat swab samples were collected from the Zhejiang Provincial Center for Disease Control and Prevention (CDC) and Chinese PLA Academy of Military Medical Sciences (AMMS). Total RNA or DNA were extracted by QIAamp MinElute Virus Spin Kit or QIAamp DNA Blood Mini Kit (Qiagen, Hilden, Germany).

### Primer and Probe Design

Gene sequences for ZEBOV, SEBOV, MARV, LFV, JUNV, MACV, RVFV, CCHFV, MP, HV, SFTSV, DENV, YFV, CHIKV, FluA, and FluB were downloaded from the nucleotide database of NCBI.^1^ The sequences were then aligned using AlignX (a component of the Vector NTI Advance 10.3.0). We designed primers to amplify the specific sequences of each pathogen mentioned above. Moreover, microarray probes ranging from 19 to 45 nucleotides were designed to detect targeted sequences of the 16 pathogens. Eventually, 29 primers and 16 were selected that accurately identified targeted pathogens. All the primers and probes were verified by BLAST^2^, and the sequences are shown in Tables 1, 2.

**TABLE 1:** The primers and their concentrations in multiplex PCR.

**Group ID**	**Primers^a^**	**Sequence (5′–3′)^b^**	**Primer conc. in multiplex PCR (mM)**	**Targeted genes**	**Pathogens**	**GenBank accession no.**
A tube	EBO-F	TCTTGAAATMAARAAACCTGAC	0.14	GP	ZEBOV and SEBOV	KM233101
	EBO-R	ACCGGGGRAAGCCYCGAATC	0.7			KC589025
	MAR-F	AATAAGAAAGTGATATTATTTGACACAA	0.1	NP	MARV	Z12132
	MAR-R	TGTTGAATTTATCCTTATCAGAATT	0.5			
	LF-F	TCAAGGACTTCAATAACA	0.1	NP	LFV	AY628201
	LF-R	CCCTGCAGACTGCAAGGATTTG	0.5			
	JUN-F	CCATCAGGTTATGTTAAGG	0.1	L	JUNV	JN801477
	JUN-R	CTATGGTGGTGGTGCTG	0.5			
	MAC-F	ATATGAAGGGAGGTGTGAACA	0.1	GP1	MACV	AY571930
	MAC-R	GCTTGAGTCAGACTTATTGAGACA	0.5			
B tube	RVF-F	GGAGAATTCCCATACCGAGTCG	0.08	NS	RVFV	KF648860
	RVF-R	GTGAAATCACTGAGAGTCATATGG	0.4			
	CCHFV-F1	TCTCAAAGAAACACGTGC	0.1	NP	CCHFV	AF362080
	CCHFV-R1	CACAAGTCCATTTCCTTTCTTGAAC	0.5			
	MAL-F1	GTAATCTTAACCATAAACTATGC	0.1	18s rRNA	MP	HQ283215
	MAL-R1	TGTCAATCCTACTCTTGTCTTA	0.5			
	HV-MFO	AAAAGTAGGTGITAYATCYTIACAATGTGG	0.16	M	HV	AF288298
	HV-MRO	GTACAICCTGTRCCIACCCC	0.8			
	SFTSV-F	AGCATGAATTCTCACGGAGC	0.12	L	SFTSV	KP202163
	SFTSV-R	CGCTCTTCAAGGTTCTGCTT	0.6			
C tube	DEN-F	TCCACCTGAGAAGGTGTAARAAATCCG	0.1	3′UTR	DENV	GQ398257
	YFV-F1	GACTCCACACATTGAATAGA	0.1	3′UTR	YFV	U54798
	FLA-R^c^	ATGTCTCCTCTACCCTCTAGWACT	0.5	3′UTR	Flavivirus	GQ398257
	CHIKV-F1	GATCATAGATGCAGTTGTAT	0.1	NSP4	CHIKV	DQ443544
	CHIKV-R2	CGCCGTACAAAGTTATGACG	0.5			
	FA-MF2	GGCCCCCTCAAAGCCGAGAT	0.1	M	Flu A	HQ664927
	FA-MR2	CAAAGCGTCTACGCTGCAGT	0.5			
	FB-F1	ATGGCCATCGGATCCTCAACTCACTC	0.1	NS	Flu B	CY099917
	FB-R1	TCATGTCAGCTATTATGGAGCTGTT	0.5			

*^*a*^F, forward primers; R, reverse primers.*

*^*b*^All the reverse primers with a biotin-labeled 5′-end.*

*^*c*^Consensus reverse primer for DENV and YFV.*

*ZEBOV, Zaire ebolavirus; SEBOV, Sudan ebolavirus; MARV, Marburg virus; LFV, Lassa fever virus; JUNV, Junin virus; MACV, Machupo virus; RVFV, Rift Valley fever virus;*

*CCHFV, Crimean-Congo hemorrhagic fever virus; MP, malaria parasite; HV, hantavirus; SFTSV, severe fever with thrombocytopenia syndrome virus; DENV, Dengue virus,*

*YFV, yellow fever virus; CHIKV, Chikungunya virus, FluA, influenza A virus; FluB, influenza B virus; JEV, Japanese encephalitis virus; HPIV, human parainfluenza virus.*

**TABLE 2:** The probes sequences for microarray.

**Pathogens**	**Probes**	**Sequence (5′–3′)^a^**
ZEBOV	ZEBO-P	GGACGGGAGCGAATGCTTAC
SEBOV	SEBO-P	TGGTGTCAGAGGCTTTCCA
MARV	MAR-P	GAGATCTCCTAGAAGGGGGTTTGCTGAC
LFV	LF-P	AGCCACAATAAATTGGGAGCAACAACTCCA
JUNV	JUN-P	CACAATCACAGTGCCGGTGGAGCCA
MACV	MAC-P	TATCAGTCTATGAACCAGAAGACCTTGGAAA
RVFV	RVF-P	GCCTTTTCAGAGACTTGTTGATCT
CCHFV	CCHFV-P	GTGTTCTCTTGAGTGCTAGCAAAATGG
MP	MAL-P	AATCAAAGTCTTTGGGTTCTGGGGCGAGTA
HV	HV-MP	GAATCCATCCTGTGGGCWGCAAGTG
SFTSV	SFTSV-P	TCCTCAGAGCTGCWTGCTCATCTC
DENV	DEN-P	CTAGCGGTTAGAGGAGACCCCTCCCTTACA GATCGCAGCAACAAT
YFV	YFV-P	CCACATGGGCTCTGCCACTGC
CHIKV	CHIKV-P	TATCAGTTGTGGTAATGTCC
Flu A	FluA-P	CTCATGGAATGGCTAAAGACAAGACCAA
Flu B	FluB-P	AATGAAGGACATTCAAAGCCAATTCGAGCAG CTGAAACTGCG
Quality control^b^	20T	TTTTTTTTTTTTTTTTTTTT

*^*a*^A repeat sequence of 12T with an amino-labeled 3****′****-end was connected to the 3****′****-end of all the probes.*

*^*b*^A repeat sequence of 20T with an amino-labeled 3****′****-end and biotin-labeled 5****′****-end was used as microarray quality control.*

### Microarray Preparation

**FIGURE 1:**
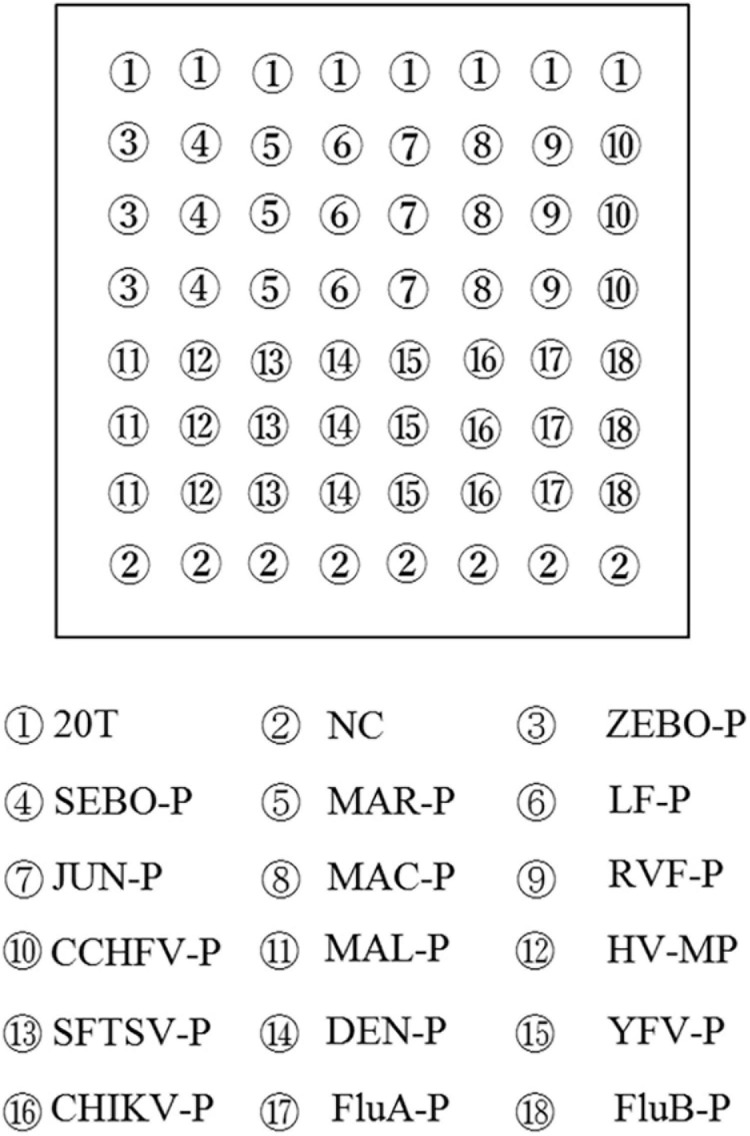
Microarray layout. Capture probes were spotted in triplicate in columns. The sequence of biotin-labeled 20 T was repeated eight times for quality control and indicated the situations of capture probes. NC, negative control.

All microarray probes were synthesized by Sangon Biotech Co., Ltd. (Shanghai, China), and a repeat sequence of 12T with an amino-labeled 3′end was connected to the 3′-end of all the probes for fixation to the aldehyde-chip surface. The microarray was composed of 10 multiwell grid holes, and each multiwell grid holes was composed of 48 points. Probes, at a 50 mM final concentration, were spotted onto the aldehyde chip after mixing with uniform proportional printing buffer [6 × saline-sodium citrate buffer (SSC), 5% glycerol, and 0.1% sodium dodecyl sulfate (SDS)]. The microarray was placed in a dryer for 24 h at room temperature, and unbound probes were removed by washing once with 0.2% SDS and once with distilled water for 30 s each at room temperature prior to use. The microarray layout is shown in Figure 1.

### RT-PCR Amplification

The 16 pathogens associated with hemorrhagic fever were divided into three groups for higher amplification efficiency. The specific fragment of each pathogen in each group was respectively amplified by a multiplex RT-PCR system. Each multiplex RT-PCR was performed in a 25-μl reaction volume containing 12.5 μl of 2 × One Step Buffer, 5 μl of template nucleic acid, and 1 μl of PrimeScript One Step Enzyme Mix (Takara Biotechnology Co., Ltd., Dalian, China). The primers for the three different RT-PCR systems are listed in Table 1. Amplifications were performed with a Veritil 96-Well Thermal Cycler PCR system (Applied Biosystems, Waltham, MA, United States) using the following conditions: 30 min at 50°C; 2 min at 94°C; 45 cycles of 20 s at 94°C, 20 s at 55°C, and 20 s at 72°C; with a final extension of 5 min at 72°C.

### Hybridization and Signal Detection

After the targeted fragments were amplified, 2 μl of each amplification product of the three reactions was mixed with 6 μl of hybridization buffer (8 × SSC, 1.2% SDS, 10% formylamine, and 10 × Denhardt’s). A total of 10 μl of hybridization mixture was added to the hybridization region on the microarray, then the chip was placed in a hybrid box, and incubated for 1 h at 45°C. Subsequently, the chip was washed once for 20 s with 1 × SSC and 0.2% SDS, followed by 0.2 × SSC, and 0.1 × SSC at room temperature.

In this assay, we introduced a chemiluminescence approach for signal detector. After hybridization and washing were complete, the chip was incubated with 15 μl of 25 nM streptavidin-horseradish peroxidase (Str-HRP, Sigma-Aldrich Co., LLC, St Louis, MO, United States) for 30 min at 37°C. Then, the chip was washed with phosphate buffered saline with 0.05% Tween 20 (PBST) for 20 s at room temperature. Subsequently, 20 μl of Chemiluminescent HRP Substrate (Millipore Corporation, Billerica, MA, United States) prepared before use was added, and the chip was scanned by Chemiluminescent Imager (Academy of Military Medical Sciences, Tianjin, China). The probe signal densities were quantified by Arrayvision 7.0. The cutoff value for each probe was calculated through the mean of the spot intensity in order to determine the signals objectively.

### Specificity and Sensitivity Evaluation

Clinical specimens that were confirmed and genotyped previously by Department of Microbiology, Zhejiang CDC, were used as positive controls for MP, HV, SFTSV, DENV, CHIKV, FluA, and FluB. The targeted genes of the remaining pathogens were synthesized by Sangon Biotech Co., Ltd. (Shanghai, China) and used for plasmid construction. Then, the RNAs were transcribed *in vitro* and also used as templates to assess the specificity of the microarray. The sensitivity of the microarray analysis was evaluated by serial 10-fold dilutions of *in vitro*-transcribed RNAs (ranging from 10^5^ to 10^1^ copies/μl). Ebola virus (Zaire) Nucleic Acid Detection Kit (real-time PCR) (Puruikang Biotech Co., Ltd., Shenzhen, China) was also used as references for sensitivity evaluation.

### Samples Detection

Sixty positive samples with nucleic acid of different pathogens (including 45 Zaire ebolavirus RNAs which were extracted from inactivated Zaire ebolavirus culture samples), were provided by the Chinese People’s Liberation Army (PLA), Academy of Military Medical Sciences (AMMS), and Zhejiang CDC, were detected by microarray analysis and commercial PCR kits. These dangerous infectious agents were handled in BSL-3 facilities of Zhejiang CDC for safety and surety. Agreement between the microarray assay and Real-Time RT-PCR assay was assessed.

## Results

### Specificity of the Microarray

**FIGURE 2:**
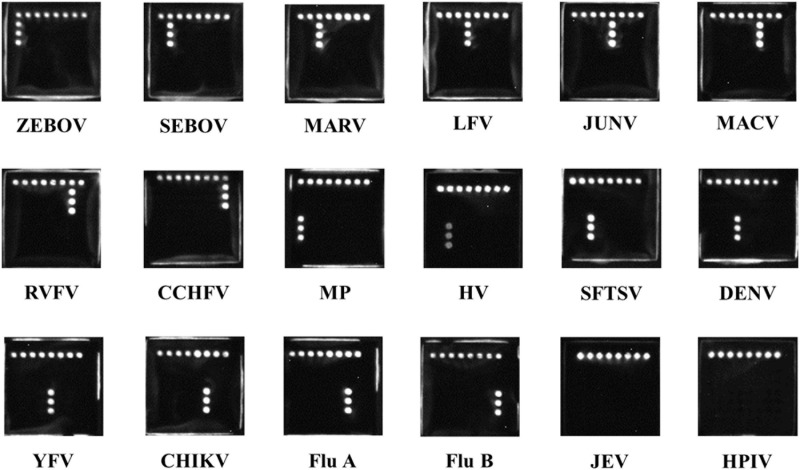
Chemiluminescence images of typical patterns for each pathogens associated with hemorrhagic fever. ZEBOV, Zaire ebolavirus; SEBOV, Sudan ebolavirus; MARV, Marburg virus; LFV, Lassa fever virus; JUNV, Junin virus; MACV, Machupo virus; RVFV, Rift Valley fever virus; CCHFV, Crimean-Congo hemorrhagic fever virus; MP, malaria parasite; HV, hantavirus; SFTSV, severe fever with thrombocytopenia syndrome virus; DENV, dengue virus; YFV, yellow fever virus; CHIKV, Chikungunya virus; FluA, influenza A virus; FluB, influenza B virus; JEV, Japanese encephalitis virus; HPIV, human parainfluenza virus.

The microarray assay was able to well distinguish nucleic acid of ZEBOV, SEBOV, MARV, LFV, JUNV, MACV, RVFV, CCHFV, MP, HV, SFTSV, DENV, YFV, CHIKV, FluA, and FluB. Also, no overlapping signal between the probes was observed. Results are shown in Figure 2.

### Sensitivity of the Microarray

**FIGURE 3:**
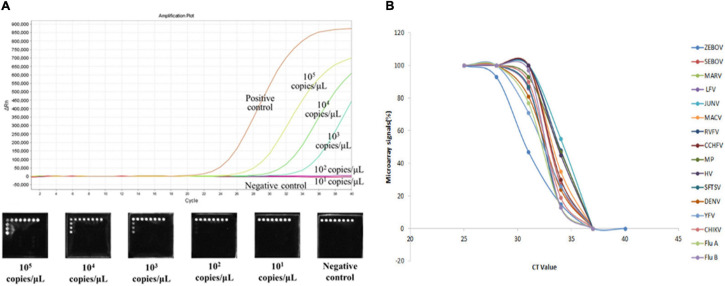
**(A)** Sensitivity comparison results of Zaire ebolavirus. The real-time RT-PCRs were amplified by the 7,500 Real-Time PCR System (ABI, Foster City, CA, United States). Five dilutions (ranging from 10^5^ to 10^1^ copies/μl) of Zaire ebolavirus *in vitro*-transcribed RNA templates were amplified to compare the sensitivity of our microarray with that of the Ebola virus (Zaire) Nucleic Acid Detection Kit (real-time PCR) (Shenzhen Puruikang Biotech Co., Ltd.). **(B)** Relationship between microarray signals and PCR cycles. Real-time RT-PCR and microarray of 16 pathogens were performed simultaneously, and the diagram display the relationship between microarray signals and PCR cycles obtained.

To determine the detection limits of the microarray assays, we prepared 10-fold serial dilutions of *in vitro* transcribed RNAs (ranging from 10^5^ to 10^1^ copies/μl). Based on the positive signals generated, the sensitivity of the assay was 10^3^ gene copies for ZEBOV, SEBOV, MARV, LFV, JUNV, RVFV, CCHFV, HV, SFTSV, DENV, YFV, CHIKV, FluA, and FluB. For MACV and MP, the detection limit was 10^2^ gene copies (Table 3). We also compared the microarray detection methods with the real-time RT-PCR method, and we discovered that our method possessed similar detection sensitivities as the real-time RT-PCR method. The sensitivity comparison results of Zaire ebolavirus genomic template are shown in Figure 3A. The relationship between microarray signals and PCR cycles were analyzed and showed in Figure 3B.

**TABLE 3:** Results for the serial dilutions of each genomic template.

**Genomic template**	**Sensitivity (copies/μ l)**				
	**10^5^**	**10^4^**	**10^3^**	**10^2^**	**10^1^**
ZEBOV	+	+	+	−	−
SEBOV	+	+	+	−	−
MARV	+	+	+	−	−
LFV	+	+	+	−	−
JUNV	+	+	+	−	−
MACV	+	+	+	+	−
RVFV	+	+	+	−	−
CCHFV	+	+	+	−	−
MP	+	+	+	+	−
HV	+	+	+	−	−
SFTSV	+	+	+	−	−
DENV	+	+	+	−	−
YFV	+	+	+	−	−
CHIKV	+	+	+	−	−
Flu A	+	+	+	−	−
Flu B	+	+	+	−	−

### Detection of Samples

The cutoff value is an index to determine hybridization results. The value was calculated by the average intensity of signals from negative pathogens and blank control plus three SD value for each probe (data not shown). A total of 60 positive samples with nucleic acid of different pathogens were tested by our microarray and commercial kits (Table 4). The ZEBOV testing results of microarray assay were 41 positive and four negative and had no cross-reactive signal with SEBOV and probes for other pathogens. The real-Ttime RT-PCR method also identified 41 samples as positive and four as negative. The results of the microarray had 100% concordance with the results of the Real-Time RT-PCR (kappa = 1.00).

**TABLE 4:** Results for all positive samples tested in the two different assays.

**Nucleic acid samples**	**No.**	**PCR**		**Microarray**	
		**+**	**−**	**+**	**−**
ZEBOV	45	41	4	41	4
Plasmodium falciparum	1	1	0	1	0
Plasmodium vivax	1	1	0	1	0
HV	2	2	0	2	0
SFTSV	4	4	0	4	0
DENV-1	1	1	0	1	0
DENV-4	1	1	0	1	0
CHIKV	1	1	0	1	0
Flu A (H1)	1	1	0	1	0
Flu A (H3)	1	1	0	1	0
Flu B (BV)	1	1	0	1	0
Flu B (Y)	1	1	0	1	0
Total	60	56	4	56	4

## Discussion

The Ebola virus is one of the greatest human infectious disease threats, with an average EVD case fatality rate approximating 50%, varing from 25 to 90% in past outbreaks (Kucharski and Edmunds, 2014; World Health Organization, 2021) according to the World Health Organization (WHO). There are numerous diseases with similar clinical syndromes (other VHFs, malaria, and influenza), their diagnosis can be a great challenge (Matua et al., 2015). Cycles of hemorrhagic fever viruses outbreak continue to be a major concern from a public health perspective and biodefense as few licensed therapeutic agents or vaccines are available. There is a persistent need for sensitive and reliable laboratory approaches for identification. A number of methods including virus culture, transmission electron microscopy, simple or multiplex PCR, IFA, and ELISA, have been utilized; however, these methods can be operationally complex, time consuming, or with low sensitivity. Most of these methods cannot meet the requirement of high throughput. Recently, serological approaches were developed for high-throughput detection of Ebola and other hemorrhagic fever viruses (Kamata et al., 2014; Wu et al., 2014). However, antibody cross-reaction between the recombinant antigens limited the application. By contrast, the advantages of the oligonucleotide microarray has distinct advantages.

In order to improve the sensitivity, we adopted the chemiluminescence detection technology widely used for ELISA (Maiolini et al., 2014), liquid hybridization assay (Hommatsu et al., 2013), and capillary electrophoresis (Jiang et al., 2013). The sensitivity of our microarray was evaluated based on cutoff values, with hybridization signals were demonstrated to be positive for samples that contained at least 10^3^ copies/μl (Table 3). These are greater detection limits labeled by fluorescence (Cy3/Cy5) (Li et al., 2009) and had similar sensitivity as the real-time RT-PCR kit (Figure 3). However, compared with other RT-PCR systems, the sensitivity of our method was much lower (Wölfel et al., 2007; Rieger et al., 2016). The reason may be due to the interaction between primers for different pathogens, and compared with other microarray systems, our microarray had a similar or better sensitivity for detecting part of these pathogens (Leski et al., 2009).

Since most of the targeted viruses are extremely dangerous, the collection of corresponding clinical sample or nucleic acid is unavailable. In order to determine the reliability of the microarray, *in vitro*-transcribed RNAs were prepared and used as templates to verify the detection results. By testing positive controls, we found that the microarray was able to distinguish all the targeted pathogens. In addition, the positive control of each pathogen could be negative control for other probes, showing an excellent specificity, without any overlapping signal between the probes (Figure 2). However, since a sufficient length of identical sequence for all 16 pathogens was not available, we had not got an internal control to verify nucleic acid templates of these pathogens extracted and the PCR reaction carried out successfully or not. This may be the disadvantage of microarray.

The GP gene of Ebola virus was chosen as an assay target for several reasons: (1) the GP protein of Ebola virus is required for entry into cells, (2) the membrane-bound GP is an important virulence factor of Ebola virus and plays a central role in the virus-mediated cytotoxicity of endothelial cells, and (3) sequence analysis of the GP genes of Ebola virus revealed that regions within this gene would be relatively conservative in evolution (Kibuuka et al., 2015; Tong et al., 2015). As more than 88% outbreaks of EVD were caused by Zaire and Sudan ebolavirus (Leroy et al., 2011; World Health Organization, 2021), we designed consensus primers for their PCR amplification and used specific probe for subtyping. The reliability of the method was also verified by detection of Ebola virus in specimens and in positive control samples.

A total of 60 samples of Ebola virus and other pathogens were tested by our microarray and compared with commercial real-time RT-PCR kits. A concordance of 100% was observed between the two methods (Table 4). However, these two methods did not positively identify four Zaire ebolavirus nucleic acid samples. These negative results may be due to low nucleic acid load or degradation of nucleic acid for inappropriate sample preservation.

## Conclusion

In conclusion, a cost-effective, multipathogen, specific, and sensitive oligonucleotide microarray assay was developed to detect Ebola virus and pathogens associated with hemorrhagic fever. This microarray was fast and high throughput, with the entire procedure, from extraction to microarray detection, could be completed within 5.5 h. The detection cost per sample was less than five US dollars. This oligonucleotide microarray will prove to be useful for treatment, prevention, surveillance, and epidemiological studies of Ebola virus.

## Data Availability Statement

The raw data supporting the conclusions of this article will be made available by the authors, without undue reservation.

## Ethics Statement

This study was approved by the Academic Committee of Zhejiang Provincial Center for Disease Control and Prevention and all experiments were carried out in accordance with the approved guidelines. The human materials used were serum or plasma specimens, blood samples and throat swab samples, and all patients provided written informed consent.

## Author Contributions

HY and YZ designed the study. HY and WY performed the experiments. ZY analyzed the data and wrote the discussion part of the manuscript. XL and HM wrote the “Results” section of the manuscript. All authors reviewed the manuscript.

## Conflict of Interest

The authors declare that the research was conducted in the absence of any commercial or financial relationships that could be construed as a potential conflict of interest.

## Publisher’s Note

All claims expressed in this article are solely those of the authors and do not necessarily represent those of their affiliated organizations, or those of the publisher, the editors and the reviewers. Any product that may be evaluated in this article, or claim that may be made by its manufacturer, is not guaranteed or endorsed by the publisher.

## Footnotes

1
http://www.ncbi.nlm.nih.gov/genomes

2
http://blast.ncbi.nlm.nih.gov/

## References

[B1] Chen E. C. Miller S. A. DeRisi J. L. Chiu C. Y. (2011). Using a pan-viral microarray assay (Virochip) to screen clinical samples for viral pathogens. *J. Vis. Exp*. 50:2536. 10.3791/2536 PMC3169278 21559002

[B2] Feldmann H. Geisbert T. W. (2011). Ebola haemorrhagic fever. *Lancet* 377 849–862. 10.1016/s0140-6736(10)60667-8 21084112 PMC3406178

[B3] Gire S. K. Goba A. Andersen K. G. Sealfon R. S. Park D. J. Kanneh L. (2014). Genomic surveillance elucidates Ebola virus origin and transmission during the 2014 outbreak. *Science* 345 1369–1372. 25214632 10.1126/science.1259657 PMC4431643

[B4] Hardick J. Woelfel R. Gardner W. Ibrahim S. (2016). Sequencing ebola and marburg viruses genomes using microarrays. *J. Med. Virol*. 88 1303–1308. 10.1002/jmv.24487 26822839

[B5] Hommatsu M. Okahashi H. Ohta K. Tamai Y. Tsukagoshi K. Hashimoto M. (2013). Development of a PCR/LDR/flow-through hybridization assay using a capillary tube, probe DNA-immobilized magnetic beads and chemiluminescence detection. *Anal. Sci*. 29 689–695. 10.2116/analsci.29.689 23842410

[B6] Huang Y. Zhu Y. Yang M. Zhang Z. Song D. Yuan Z. (2014). Nucleoprotein-based indirect enzyme-linked immunosorbent assay (indirect ELISA) for detecting antibodies specific to Ebola virus and Marbug virus. *Virol. Sin*. 29 372–380. 10.1007/s12250-014-3512-0 25547682 PMC8206289

[B7] Jiang J. Zhao S. Huang Y. Qin G. Ye F. (2013). Highly sensitive immunoassay of carcinoembryonic antigen by capillary electrophoresis with gold nanoparticles amplified chemiluminescence detection. *J. Chromatogr. A*. 1282 161–166. 10.1016/j.chroma.2013.01.066 23422894

[B8] Kamata T. Natesan M. Warfield K. Aman M. J. Ulrich R. G. (2014). Determination of specific antibody responses to the six species of ebola and marburg viruses by multiplexed protein microarrays. *Clin. Vaccine Immunol*. 21 1605–1612. 10.1128/cvi.00484-14 25230936 PMC4248775

[B9] Katoski S. E. Meyer H. Ibrahim S. (2015). An approach for identification of unknown viruses using sequencing-by-hybridization. *J. Med. Virol*. 87 1616–1624. 10.1002/jmv.24196 25976068

[B10] Kibuuka H. Berkowitz N. M. Millard M. Enama M. E. Tindikahwa A. Sekiziyivu A. B. (2015). Safety and immunogenicity of Ebola virus and Marburg virus glycoprotein DNA vaccines assessed separately and concomitantly in healthy Ugandan adults: a phase 1b, randomised, double-blind, placebo-controlled clinical trial. *Lancet* 385 1545–1554. 10.1016/s0140-6736(14)62385-0 25540891

[B11] Kolquist K. A. Chultz R. A. Furrow A. Brown T. C. Han J. Y. Campbell L. J. (2011). Microarray-based comparative genomic hybridization of cancer targets reveals novel, recurrent genetic aberrations in the myelodysplastic syndromes. *Cancer Genet*. 204 603–628. 10.1016/j.cancergen.2011.10.004 22200086

[B12] Kucharski A. J. Edmunds W. J. (2014). Case fatality rate for Ebola virus disease in west Africa. *Lancet* 384:1260. 10.1016/s0140-6736(14)61706-2 25260235

[B13] Leroy E. M. Gonzalez J. P. Baize S. (2011). Ebola and Marburg haemorrhagic fever viruses: major scientific advances, but a relatively minor public health threat for Africa. *Clin. Microbiol. Infect*. 17 964–976. 10.1111/j.1469-0691.2011.03535.x 21722250

[B14] Leski T. A. Lin B. Malanoski A. P. Wang Z. Long N. C. Meador C. E. (2009). Testing and validation of high density resequencing microarray for broad range biothreat agents detection. *PLoS One* 4:e6569. 10.1371/journal.pone.0006569 19668365 PMC2719057

[B15] Li X. Qi X. Miao L. Wang Y. Liu F. Gu H. (2009). Detection and subtyping of influenza A virus based on a short oligonucleotide microarray. *Diagn. Microbiol. Infect. Dis*. 65 261–270. 10.1016/j.diagmicrobio.2009.07.016 19733996

[B16] Liu L. Sun Y. Kargbo B. Zhang C. Feng H. Lu H. (2015). Detection of Zaire Ebola virus by real-time reverse transcription-polymerase chain reaction, Sierra Leone, 2014. *J. Virol. Methods* 222 62–65. 10.1016/j.jviromet.2015.05.005 26025458

[B17] Maiolini E. Ferri E. Pitasi A. L. Montoya A. Di Giovanni M. Errani E. (2014). Bisphenol A determination in baby bottles by chemiluminescence enzyme-linked immunosorbent assay, lateral flow immunoassay and liquid chromatography tandem mass spectrometry. *Analyst* 139 318–324. 10.1039/c3an00552f 24223419

[B18] Martin P. Laupland K. B. Frost E. H. Valiquette L. (2015). Laboratory diagnosis of Ebola virus disease. *Intens. Care Med*. 41 895–898. 10.1007/s00134-015-3671-y 25636586

[B19] Marty A. M. Jahrling P. B. Geisbert T. W. (2006). Viral hemorrhagic fevers. *Clin. Lab. Med*. 26 345–386. 16815457 10.1016/j.cll.2006.05.001

[B20] Matua G. A. Van der Wal D. M. Locsin R. C. (2015). Ebolavirus and Haemorrhagic Syndrome. *Sultan. Qaboos Univ. Med. J*. 15 e171–e176. 26052448 PMC4450778

[B21] Maxmen A. (2020). World’s second-deadliest Ebola outbreak ends in Democratic Republic of the Congo. *Nature* 10.1038/d41586-020-01950-0 34172954

[B22] Mazzatti D. Lim F. L. O’Hara A. Wood I. S. Trayhurn P. A. (2012). microarray analysis of the hypoxia-induced modulation of gene expression in human adipocytes. *Arch. Physiol. Biochem*. 118 112–120. 10.3109/13813455.2012.654611 22352407

[B23] Rieger T. Kerber R. El Halas H. Pallasch E. Duraffour S. Günther S. (2016). Evaluation of RealStar reverse transcription-polymerase chain reaction kits for Filovirus detection in the laboratory and field. *J. Infect. Dis*. 214 S243–S249. 27549586 10.1093/infdis/jiw246 PMC5050472

[B24] Tong Y. G. Shi W. F. Liu D. Qian J. Liang L. Bo X. C. (2015). Genetic diversity and evolutionary dynamics of Ebola virus in Sierra Leone. *Nature* 524 93–96. 25970247 10.1038/nature14490 PMC10601608

[B25] Wölfel R. Paweska J. T. Petersen N. Grobbelaar A. A. Leman P. A. Hewson R. (2007). Virus detection and monitoring of viral load in Crimean-Congo hemorrhagic fever virus patients. *Emerg. Infect. Dis*. 13 1097–1100. 10.3201/eid1307.070068 18214191 PMC2878241

[B26] World Health Organization (2021). *Ebola Virus Disease.* Available online at: https://www.who.int/news-room/fact-sheets/detail/ebola-virus-disease (accessed February 27, 2021).

[B27] Wu W. Zhang S. Qu J. Zhang Q. Li C. Li J. (2014). Simultaneous detection of IgG antibodies associated with viral hemorrhagic fever by a multiplexed Luminex-based immunoassay. *Virus Res*. 187 84–90. 10.1016/j.virusres.2013.12.037 24631566

[B28] Zhang Y. Liu Q. Wang D. Chen S. Wang S. (2013). Simultaneous detection of oseltamivir- and amantadine-resistant influenza by oligonucleotide microarray visualization. *PLoS One* 8:e57154. 10.1371/journal.pone.0057154 23451169 PMC3579783

